# Needle aspirate PTH in diagnosis of primary hyperparathyroidism due to intrathyroidal parathyroid cyst

**DOI:** 10.1530/EDM-13-0019

**Published:** 2013-07-01

**Authors:** Deep Dutta, Chitra Selvan, Manoj Kumar, Saumik Datta, Ram Narayan Das, Sujoy Ghosh, Satinath Mukhopadhyay, Subhankar Chowdhury

**Affiliations:** 1 Department of Endocrinology and MetabolismIPGMER and SSKM HospitalRoom-9A, 4th Floor Ronald Ross Building, 244 AJC Bose Road, Calcutta, 700020India; 2 Department of PathologyIPGMER and SSKM HospitalRoom-9A, 4th Floor Ronald Ross Building, 244 AJC Bose Road, Calcutta, 700020India

## Abstract

Parathyroid cysts are rare (0.8–3.41% of all parathyroid lesions) and usually arise secondary to cystic degeneration of parathyroid adenomas. Intrathyroidal parathyroid cysts are extremely rare with only three cases reported till date. We present a 24-year-old female with clinical and biochemical features of primary hyperparathyroidism (PHPT; Ca^2^^+^: 12.1 mg/dl; intact parathyroid hormone (iPTH): 1283 pg/ml) and poor radiotracer uptake with minimal residual uptake in the left thyroid lobe at 2 and 4 h on Tc^99m^ sestamibi imaging. Neck ultrasonography (USG) revealed 0.6×1 cm parathyroid posterior left lobe of thyroid along with 22×18 mm simple thyroid cyst. USG-guided fine-needle aspiration (FNA) and needle tip iPTH estimation (FNA-iPTH) from parathyroid lesion was inconclusive (114 pg/ml), necessitating FNA of thyroid cyst, which revealed high iPTH (3480 pg/ml) from the aspirate. The patient underwent a left hemithyroidectomy. A >50% drop in serum iPTH 20 min after left hemithyroidectomy (29.4 pg/ml) along with histopathology suggestive of intrathyroidal cystic parathyroid adenoma (cystic lesion lined by chief cell variant parathyroid cells without any nuclear atypia, capsular or vascular invasion surrounded by normal thyroid follicles) confirmed that the parathyroid cyst was responsible for PHPT. This report highlights the importance of FNA-iPTH in localizing and differentiating a functional parathyroid lesion from nonfunctional tissue in PHPT.

**Learning points:**

- Fine-needle aspiration from suspected parathyroid lesion and needle tip iPTH (FNA-iPTH) estimation from the saline washing has an important role in localizing primary hyperparathyroidism (PHPT).
- FNA-iPTH estimation may help in differentiating functional from nonfunctional parathyroid lesion responsible for PHPT.
- iPTH estimation from aspirate of an intrathyroid cyst is helpful in differentiating intrathyroidal parathyroid cyst from thyroid cyst.

## Background

Primary hyperparathyroidism (PHPT) is not an uncommon disorder with a prevalence of 4–30 per 100 000 population. It occurs most commonly in the sixth decade, with parathyroid adenoma being the underlying pathology in the vast majority of cases (75–80%) (1). In contrast, parathyroid cysts are rare (0.8–3.41% of all parathyroid lesions) with <300 cases reported (1). Parathyroid cysts are usually secondary to cystic degeneration of parathyroid adenomas (1–2% of PHPT) or very rarely true parathyroid cysts (simple cysts) (2). True parathyroid cysts are clinically silent in contrast to cysts due to degeneration of parathyroid adenomas that are usually functional (associated PHPT) (2).

Intrathyroidal parathyroid cysts are extremely rare with only three cases reported till date (3) (4) (5). We present a female with functional intrathyroid parathyroid cyst causing PHPT.

## Case report

**Figure 1:**
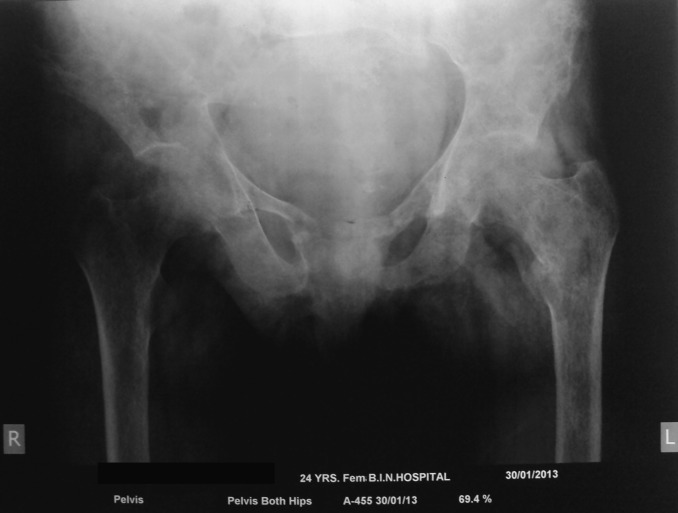
X-ray of the pelvis showing multiple extensive lytic lesions involving the iliac bones, pubic rami, and greater trochanters of the femur along with cortical thinning and increased lucency of the bones consistent with osteitis fibrosa cystica (Von Recklinghausen's disease of bone).

PHPT was diagnosed in a 24-year-old female presenting with diffuse bone pains and proximal weakness for one and a half years, osmotic symptoms and polyuria for 6 months, along with biochemical evidence of hypercalcemia, hypophosphatemia, elevated alkaline phosphate, intact parathyroid hormone (iPTH), severe osteoporosis, and skeletal imaging suggestive of PHPT (Table 1 and Fig. 1). Examination was significant for severe proximal weakness in bilateral lower limbs and 2×2 cm palpable nodule in lower left lobe of thyroid.

**Table 1:** Biochemical profile of the patient pre- and post-hemithyroidectomy

**Parameter**	**Baseline** (December 2012)	**Day of surgery** (April 2013)	**Postoperative day 2**	**Postoperative day 7**
Calcium (mg/dl) (8.6–10.8)	12.1	11.4	7.4	8.7
Phosphorus (mg/dl) (3.5–5)	1.5	2.3	3.5	3.9
ALP (U/l) (38–136)	1856			
Bone fraction ALP (μg/l) (3–19)	120			
Albumin (mg/dl) (3.5–4.2)	3.9	3.8	3.7	3.8
25OHD (ng/ml) (30–100)	11.79	37.6^a^		
iPTH (pg/ml) (7–65)	1283	1054 (29.4)^b^		33
Creatinine (mg/dl)	0.6	0.8		0.8
SGPT (U/l)	18			
Prolactin (ng/ml) (0–20)	4.49			
IGF1 (ng/ml) (116–358)	154			
Free T_4_ (ng/dl) (0.9–1.8)	1.25			
TSH (μU/ml) (0.4–4.2)	3.12			
BMD (g/cm^2^)				
Spine	0.513 (−5.6)^c^			
Left femur neck	0.308 (−5.6)^c^			
Right femur total	0.363 (−5.3)^c^			

ALP, alkaline phosphate; 25OHD, 25-hydroxyvitamin-D; iPTH, intact parathyroid hormone; SGPT, serum glutamic pyruvic transaminase; BMD, bone mineral density.

a Patient received cholecalciferol sachets 60 000 U (DRISE, USV, Mumbai, India) once weekly for 8 weeks after the baseline investigations; prolactin and IGF1 were done to rule out multiple endocrine neoplasia-1.

b Value in parentheses represents serum iPTH 20 min after left hemithyroidectomy.

c Value in parentheses represent *T*-score.

## Investigation

**Figure 2:**
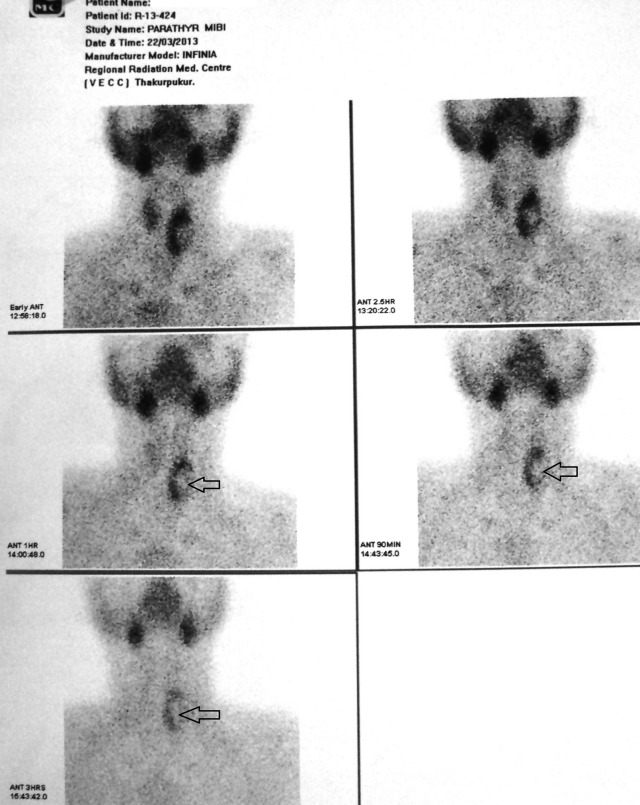
Tc^99m^ sestamibi scan did not reveal any functional parathyroid adenoma. Poor radiotracer uptake in the lower pole and lateral aspect of left lobe of thyroid suggestive of cold nodule (black arrow).

**Figure 3:**
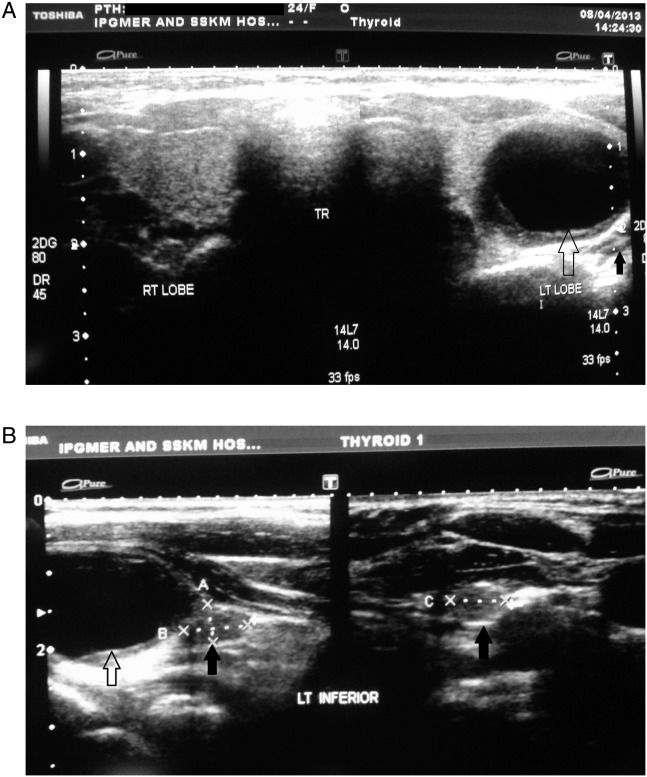
(A) Ultrasonography of the neck revealed simple cyst in the thyroid parenchyma corresponding to the palpable nodule in the left lobe (hollow black arrow). A 0.6×1.0 cm hypoechoic well-demarcated elliptic lesion with smooth borders and a hyperechoic line on the ventral surface were also noted posterior to thyroid (solid black arrow). (B) Ultrasonography neck with better characterization of the hypoechoic elliptic lesion posterior to left lobe thyroid suggestive of parathyroid adenoma (solid black arrow). The intrathyroidal simple cyst can be noted adjacent to it (hollow black arrow).

Tc^99m^ sestamibi imaging for localizing parathyroid lesion revealed poor radiotracer uptake with minimal residual uptake at 2 and 4 h in the lower left lobe of thyroid (Fig. 2). Neck ultrasonography (USG) revealed 22×18 mm homogenous cystic lesion in lower pole of left lobe of thyroid suggestive of simple thyroid cyst along with 0.6×1.0 cm hypoechoic well-demarcated elliptic lesion with smooth borders and a hyperechoic line on the ventral surface, posterior to thyroid, suggestive of parathyroid adenoma (Fig. 3). She underwent USG-guided fine-needle aspiration (FNA) of the suspected parathyroid adenoma followed by iPTH measurement from the needle washing (FNA-iPTH) using chemiluminescent microparticle immunoassay (Immulite-1000, Siemens, Gwynedd, UK; analytical sensitivity: 2 ng/ml; intra- and interassay coefficient of variation 4 and 6% respectively). FNA-iPTH was 114 pg/ml, which was lower than serum iPTH (Table 1), making this lesion unlikely to be responsible for PHPT. This led to USG-guided aspiration of the thyroid cyst. Six milliliters of serous fluid were aspirated, iPTH measurement from which revealed a level of 3480 pg/ml.

## Management and clinical course

The patient underwent a left hemithyroidectomy. Her preoperative (on the day of surgery) and 20-min post-hemithyroidectomy iPTH were 1054 and 29.4 pg/ml respectively, indicating surgical cure of PHPT. Thereafter, left inferior parathyroid was resected. Left superior parathyroid could not be localized during surgery.

**Figure 4:**
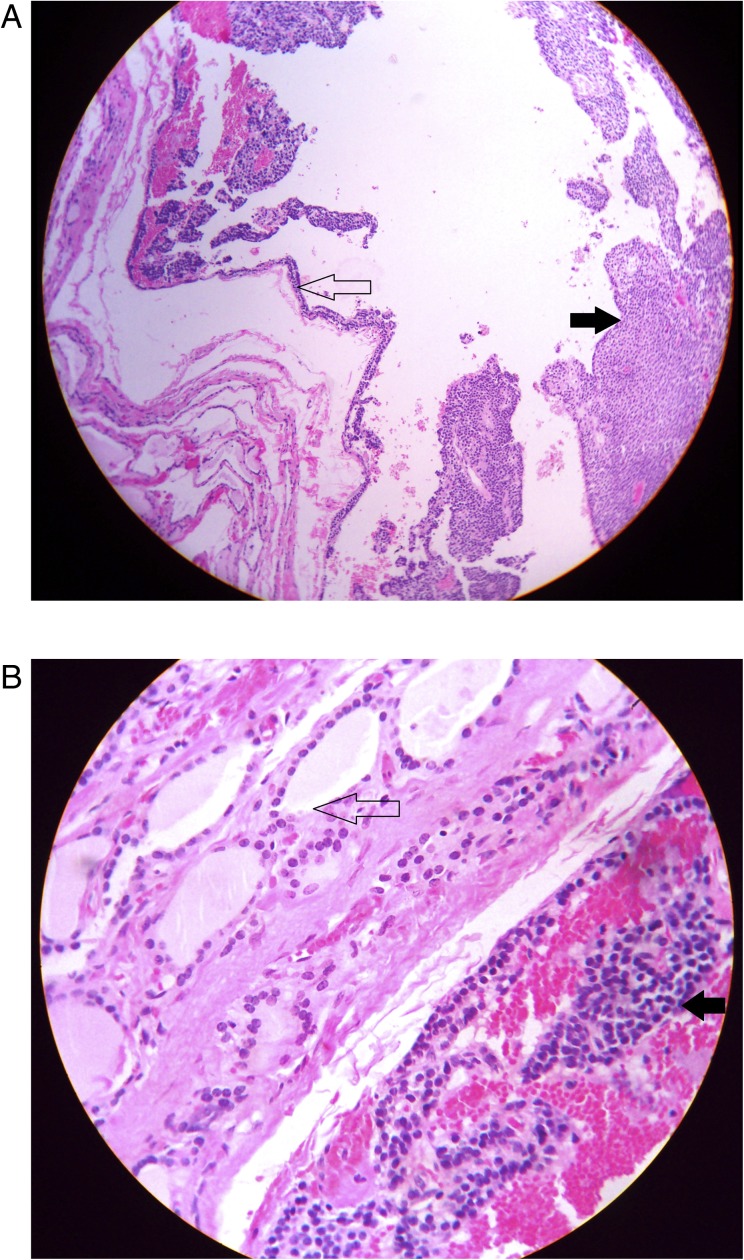
(A) Eosin and hematoxylin staining of left hemithyroidectomy specimen showing the cyst wall lined by parathyroid cells (hollow black arrow), along with sheets of parathyroid cells without any capsular or vascular invasion (solid black arrow). A few thyroid follicles can also been seen at 7–9 o' clock position. (B) Higher magnification showing highly cellular homogenous cell population arranged in nests suggestive of chief cell type of parathyroid adenoma (solid black arrow) with adjacent thyroid follicles (hollow black arrow) confirming the intrathyroid location of the parathyroid cyst.

**Figure 5:**
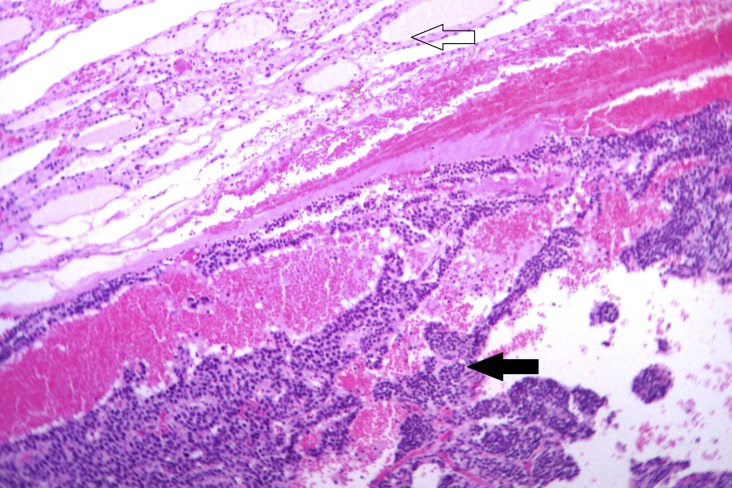
Eosin and hematoxylin staining showing sheets of parathyroid cells (solid black arrow) with adjacent thyroid follicles (hollow black arrow) confirming the intrathyroidal location of the parathyroid cyst.

Postoperative tetany and hypocalcemia were documented, which resolved with therapy of calcium and calcitriol. Histopathology of surgical specimen revealed a cystic lesion lined by chief cell variant parathyroid cells without any nuclear atypia, capsular or vascular invasion, surrounded by normal thyroid follicular cells, suggestive of intrathyroidal cystic parathyroid adenoma (Figs 4 and 5). When last evaluated, 7 weeks after surgery, she was asymptomatic and off calcium and calcitriol.

## Discussion

Ectopic parathyroid adenoma is a well-recognized entity and can occur anywhere from the upper part of the neck to the mediastinum. It is believed to be due to abnormal migration of parathyroid cells during embryogenesis. Ectopic superior parathyroids have been most commonly observed above intersection between recurrent laryngeal nerve and superior thyroid artery, followed by cricothyroid junction, posterior to upper pole of thyroid and rarely in retropharyngeal, retroesophageal, and intrathyroidal regions (5). In contrast, ectopic inferior parathyroids have been documented most commonly between lower pole of thyroid and thymus, followed by intra-thymus and in anterior mediastinum (5).

Accurate preoperative localization using Tc^99m^ sestamibi imaging has played a major role in reducing operative morbidity in PHPT. Tc^99m^ sestamibi can accurately localize 60–80% of single parathyroid adenomas accounting for 75–80% of PHPT (6). USG is a useful adjunct to Tc^99m^ sestamibi having 51–78 and 67–96% sensitivity and specificity in anatomic localization of parathyroid adenoma (6). Normal parathyroids are usually elliptic discoid, appear fatty, 4–5 mm length, 2–4 mm breadth, and 1–2 mm height, posterior to thyroid, with echogenicity similar to surrounding tissue making them undetectable on USG (7). Increased size, smooth borders, development of hypoechogenicity due to increased cellularity, with hyperechogenicity of capsule, and reduction of fatty content makes parathyroid visible on USG (7). Hypoechoic halo seen around thyroid nodules (due to compressed blood vessels) are absent in parathyroid nodules, which aid in their differentiation (7).

FNA biopsy along with FNA-iPTH measurement has been suggested to be useful in localizing parathyroid adenomas by providing simultaneous cytological and biochemical evidence, especially in the setting of inconclusive neck USG and Tc^99m^ sestamibi imaging (8). FNA-iPTH is considered positive and diagnostic of parathyroid adenoma if FNA-iPTH level is higher than serum iPTH of the patient (9). FNA-iPTH was negative in our patient. However, increased iPTH (3480 pg/ml) from cyst aspirate confirmed it to be parathyroid cyst and not thyroid cyst as initially assumed. A >50% drop of serum iPTH following left hemithyroidectomy along with histology showing intrathyroid cyst lined by parathyroid cells with adjacent thyroid follicles confirmed that the parathyroid cyst was intrathyroidal and responsible for PHPT.

Functional intrathyroid parathyroid cyst causing PHPT was first reported in a 54-year man with nephrolithiasis and positive Tc^99m^ sestamibi imaging (3). FNA from the cyst revealed parathyroid cells, and there was resolution of PHPT following right hemithyroidectomy (3). In contrast, increased FNA-iPTH from an incidentally detected intrathyroid cyst, during USG evaluation of a multinodular goiter, lead to diagnosis of nonfunctional intrathyroidal parathyroid cyst in a 29-year-old asymptomatic female with papillary microcarcinoma of thyroid (4). Histopathological evaluation of left hemithyroidectomy, done for left-sided neck lump and simple thyroid cyst in a 19-year-old female, was diagnosed to be nonfunctional intrathyroidal parathyroid cyst (5). Pacini *et al*. (10) reported seven cases of incidental parathyroid cyst during evaluation of 112 individuals with cysts in the neck (6.2%), diagnosed by FNA-iPTH with mean levels ranging from 6780 to 416 650 pg/ml.

Our patient probably had cystic degeneration of parathyroid adenoma. Retrospectively, it may be speculated that the parathyroid localized on USG posterior to thyroid was an enlarged nonfunctional left inferior parathyroid, and the functional intrathyroid parathyroid cyst was most likely an ectopic left superior parathyroid. This report intends to highlight the importance of needle tip FNA-iPTH estimation in localizing a functional parathyroid lesion, as well as ruling out a nonfunctional parathyroid lesion.

## Patient consent

Written informed consent was obtained from the patient/patient's mother for publication of this case report.

## Author contribution statement

The patient was initially evaluated and admitted under the care of S Ghosh. S Chowdhury and S Mukhopadhyay reviewed the patient and took treatment decisions. Investigations were done by M Kumar and S Datta. The ultrasonography-guided fine-needle aspiration was done by D Dutta and C Selvan. Histopathological evaluation was done by R N Das. The initial draft of the manuscript was prepared by D Dutta. All authors contributed equally in editing the draft and preparation of the final manuscript.
